# A Paratesticular Angiolipoma: A Case Report of a Rare Benign Scrotal Mass and Review of Literature

**DOI:** 10.1155/2019/1478573

**Published:** 2019-06-20

**Authors:** Mohamed Ali Nouioui, Tarek Taktak, Seif Mokadem, Faten Gargouri, Houssem Mediouni, Ramzi Khiari, Samir Ghozzi

**Affiliations:** ^1^Department of Urology, Military Hospital of First Instruction of Tunis, Tunisia; ^2^Department of Pathology, Military Hospital of First Instruction of Tunis, Tunisia

## Abstract

We report a rare case of paratesticular angiolipoma in a young male. The patient is a 21-year-old male who presented with a palpable firm right intrascrotal mass of 21 mm. Ultrasound findings demonstrated that it is a solid mass. Under the diagnosis of an intrascrotal solid mass, a right inguinal radical orchiectomy was performed. Histopathological examination concluded to a paratesticular angiolipoma. Angiolipoma is a rare benign form of paratesticular tumour and its diagnosis is based on histological findings of the surgical specimen with no recurrence risk. This mesenchymal tumour should be distinguished from liposarcoma, which has malignant or aggressive clinical course.

## 1. Introduction

Angiolipoma is an extremely rare form of paratesticular tumour observed in urologic clinical practice [1]. A search of the Medline database revealed few rare cases of such lesion, usually confirmed by the microscopic pathologic evaluation of the surgical specimen. Tumours occurring in the paratesticular region may be clinically indistinguishable from intratesticular tumours, thus resulting in initial misdiagnosis. The preoperative distinction between the benign and malignant lesion is rarely made which results in difficulty in diagnosis and management [2] such as in the case reported herein.

## 2. Case Presentation

A 21-year-old male with a history of one episode of right orchitis well treated with antibiotics two years ago presented to the urology outpatient department with a painless swelling of the right hemiscrotum without any associated other symptoms. Local examination revealed a palpable firm right testicular mass with atrophy of the whole testis. The left testis was palpated in the scrotum and is of normal size with no suspicious mass. His laboratory findings including Germ Cell Tumour serum markers were within the normal range (Alpha-fetoprotein (AFP): 0,86 *μ*g/L, Beta human chorionic gonadotrophin (*β*-HCG) < 0,5 mIU/mL, and lactate dehydrogenase (LDH): 241 UI/ml).

**Figure 1:**
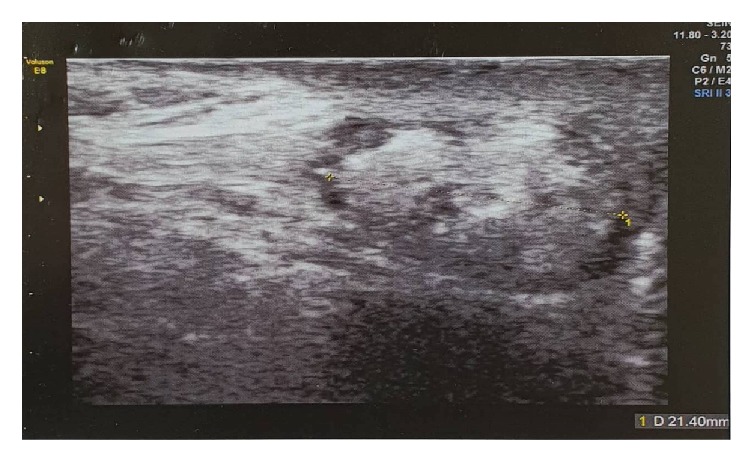
US demonstrating a solid 21 mm mass within the right epididymis.

**Figure 2:**
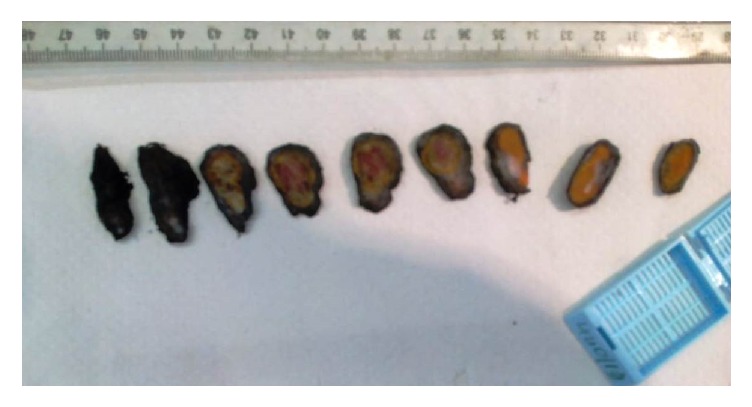
Sectioning of the specimen revealing a single well circumscribed nodular tissue.

**Figure 3:**
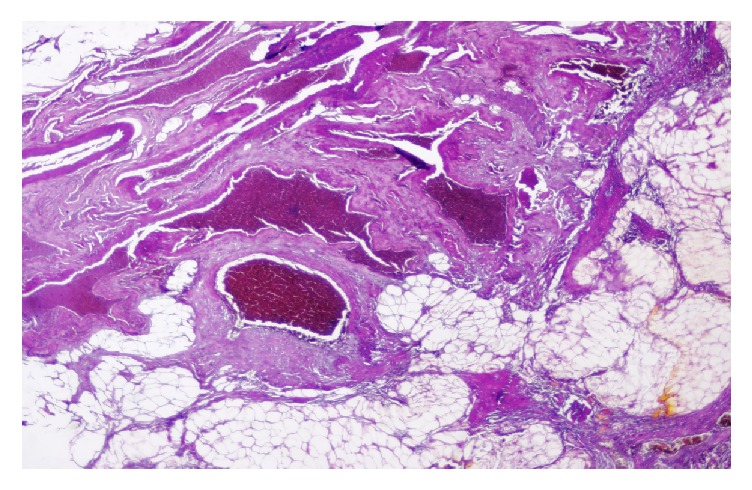
Microscopic examination showing a mature lobulated adipose tissue and numerous dilated and thicken walled blood vessels.

Scrotal ultrasound (Figure 1) demonstrated a suspicious solid well-defined mass within the right epididymis, measuring 21 x 14 mm. The mass was slightly heterogeneous with hyperechoic appearance and regular smooth contour. Distinct from the lesion, the right testis appeared smaller in size measuring 23 x 12 x 15 mm. Imaging of the contralateral scrotum revealed no anomaly. A radical right orchiectomy was performed via an inguinal incision. Recovery was uneventful. The surgical specimen consisted of the right testis measuring 3.5x2.5x1 cm in size and of the spermatic cord measuring 5 cm in length. Sectioning of the specimen (Figure 2) revealed a single well circumscribed nodular tissue with multiple foci of hemorrhage. Microscopic examination of the nodular tissue (Figure 3) showed an admixture of mature lobulated adipose tissue and numerous dilated and thicken walled blood vessels with no signs of malignancies such as germ cell atypia or intraepithelial germ cell neoplasm. Thus a diagnosis of paratesticular angiolipoma was made.

## 3. Discussion

The paratesticular region is a complex anatomical area which includes the contents of the spermatic cord, testicular tunics, epididymis, and vestigial remnants. Histogenetically, this area is composed of a variety of epithelial, mesothelial, and mesenchymal elements and neoplasms arising from this region, therefore form a heterogeneous group of tumours with different behavioural patterns [3]. While 95% of intratesticular lesions are malignant, most of paratesticular masses are benign [4]. Lipoma accounts for approximately 90% of benign paratesticular soft tissues tumours. Variants of lipoma, including fibrolipoma and angiolipoma, may arise in the testis such in our case [5]. Usually the most common clinical manifestations include vague scrotal discomfort or heaviness leading to a physical examination that discovers a painless firm scrotal mass. Because of the availability, ease of use, and high resolution of ultrasonography (US), it is the imaging modality of choice for testicular pathologies and characterisation of testicular masses [6] with high sensitivity regarding the detection, localization, and sizing of such lesions. However, it shows low specificity in differentiating benign from malignant type [7]. This makes an inguinal testicular exploration necessary because the diagnosis of paratesticular angiolipoma can only be made by histological evaluation of the surgical specimen. One of the top differential diagnoses of angiolipoma is well differentiated liposarcoma which is a malignant mesenchymal neoplasm showing adipocytic differentiation with completely different management recommendations and prognosis.

Less than 5% of soft tissue sarcomas arise from the genitourinary tract, accounting for only 1–2% of all malignant genitourinary tumours [8].

Liposarcoma accounts for 20 to 56% of sarcomas at this anatomical site in adults and most paratesticular liposarcomas are well differentiated [2, 9].

Due to the rarity of this type of tumour, clinical research regarding this disease is difficult.

Both benign lipoma and liposarcoma present as a nonspecific painless paratesticular, sometimes inguinal, firm mass.

On gross examination, lipoma-like well differentiated liposarcoma resembles mature fat which makes it difficult for initial intraoperative assessment by surgeons, thus mistreated with initial marginal resection of the lesion which is largely insufficient for such an aggressive tumour. Histologic features are important in the assessment of these lesions because well differentiated liposarcoma consists of mature adipocytes with marked variation in cell size, nuclear atypia such as enlarged hyperchromatic nuclei, and a variable number of scatted lipoblasts, all not found in benign adipocytic lesions [10].

Immunohistochemically, the most useful diagnostic markers are MDM2 and CDK4, which allow distinguishing well differentiated liposarcoma from benign lipomatous lesions [11].

The clinical course of well differentiated liposarcoma has tendency for local recurrence after inadequate resection, whereas distant spread is rare but common for high grade tumours. Local recurrence is often repeated and may involve the inguinal canal, pelvis, and scrotum [12, 13].

Thus, the recommended treatment policy for liposarcoma includes aggressive local management consisting of surgery and RT for most patients [14].

Appropriate surgery consists of radical inguinal orchiectomy and wide excision of the prior scar and tumour bed if initially excised including removal of all the soft tissues in the inguinal canal with high ligation of the spermatic cord at the level of the inguinal ring. Hemiscrotectomy should be performed in cases where the scrotum was violated by prior biopsy or excision or if there was direct involvement of the scrotum by tumour [12].

However, angiolipoma has a favourable prognosis with no recurrence rate if well excised.

## 4. Conclusion

Angiolipoma is an uncommon benign tumour that is rarely found in the scrotum but need to be considered when assessing a patient with scrotal mass. Ultrasound findings can be indecisive. An inguinal surgical approach provides a suitable exposure for complete resection. Histopathological examination is the only way to provide the definitive diagnosis to distinguish it from a lipoma-like well differentiated liposarcoma, thus determining the appropriate course of treatment.
